# Unidirectional animal-to-human transmission of methicillin-resistant *Staphylococcus aureus* ST398 in pig farming; evidence from a surveillance study in southern Italy

**DOI:** 10.1186/s13756-019-0650-z

**Published:** 2019-11-21

**Authors:** Mattia Pirolo, Daniela Visaggio, Angela Gioffrè, Irene Artuso, Monica Gherardi, Grazia Pavia, Pasquale Samele, Lucia Ciambrone, Rossella Di Natale, Giovanna Spatari, Francesco Casalinuovo, Paolo Visca

**Affiliations:** 1 Department of Science, Roma Tre University, Viale G. Marconi 446, 00146 Rome, Italy; 2 Department of Medicine, Epidemiology, Workplace and Environmental Hygiene, Lamezia Terme Research Centre, INAIL – National Institute for Insurance against Accidents at Work, Lamezia Terme, Italy; 3 Department of Medicine, Epidemiology, Workplace and Environmental Hygiene, Monte Porzio Catone Research Centre, INAIL – National Institute for Insurance against Accidents at Work, Rome, Italy; 4 Istituto Zooprofilattico Sperimentale del Mezzogiorno, Catanzaro, Italy; 5 Department of Biomedical Sciences, Dental, Morphological and Functional Investigations, University of Messina, Messina, Italy

**Keywords:** Antimicrobial resistance, Biological risk, Epidemiological typing, Farming, Livestock, MRSA, Occupational medicine, *Staphylococcus aureus*, Swine, Zoonosis

## Abstract

**Background:**

Livestock-associated methicillin-resistant *Staphylococcus aureus* (MRSA) belonging to clonal complex 398 is recognized as an occupational hazard for workers employed in intensive animal husbandry, especially in the swine-breeding chain. In this study, we compared the prevalence and epidemiological type of MRSA isolates from swine and farm workers in a large area of southern Italy.

**Methods:**

Between January and March 2018, 88 workers from 32 farms where we had previously performed a survey for MRSA colonization of farmed pigs, were sampled by nasal swabbing. A follow-up investigation was conducted on seven workers 1 year after primary screening. MRSA isolates were characterized by MLST, *spa* and SCC*mec* typing, and tested for susceptibility to 15 antimicrobials. Epidemiological correlations between human and swine MRSA isolates were supported by Rep-MP3 and RAPD PCR fingerprinting, and whole-genome sequencing.

**Results:**

The overall colonization rate of MRSA in swine farm workers was 21.6%, being significantly higher in intensive farms and in workers with direct animal contact. All human MRSA isolates were multi-drug resistant, belonged to the ST398 livestock clade, and did not carry Panton-Valentine leukocidin and enterotoxin genes. Notably, 94.1% of human MRSA isolates belonged to the same epidemiological type as swine MRSA isolates from the corresponding farm. Persistent MRSA carriage was documented in some workers 1 year after primary sampling.

**Conclusions:**

We report a high prevalence of MRSA among swine farm workers, with higher colonization rates associated with intensive breeding and animal exposure. Our findings suggest unidirectional animal-to-human transmission of LA-MRSA and denote the high zoonotic transmissibility of the ST398 livestock clade.

## Background

*Staphylococcus aureus* is a skin and mucosal commensal of humans and animals, and an important human pathogen involved in various infections, ranging from localized to life-threatening invasive diseases. Although human colonization varies with geographic location, seasonality, age and sex, ca. 30% individuals are nasal carriers of *S. aureus* [1].

*S. aureus* rapidly adapts to the selective pressure imposed by antimicrobial therapy, and methicillin-resistant *S. aureus* (MRSA) has spread in both healthcare (hospital-associated MRSA, HA-MRSA) and community (community-associated MRSA, CA-MRSA) settings [2]. Over the last decade, particular MRSA lineages also emerged in livestock animals (livestock-associated MRSA, LA-MRSA), with variable prevalence in different geographic regions. The predominant LA-MRSA clonal complex (CC) in Europe and North America is CC398, with the majority of the strains belonging to sequence type (ST) 398 (reviewed by ref. [3]), whereas ST(CC)9 LA-MRSA predominates in Asia [2].

LA-MRSA ST398 is recognized as an occupational hazard for people working in the intensive animal husbandry or living in high-density livestock production areas ([4, 5] reviewed by ref. [3]). Several studies have reported a positive correlation between human colonization by LA-MRSA and intensity of animal contact, especially in farmers, abattoir workers, and veterinarians [6–10]. Colonization by MRSA is a prerequisite to human infection, and cases of severe infections caused by LA-MRSA ST398 have been reported, resembling those caused by CA-MRSA ([11, 12] reviewed by ref. [3]).

LA-MRSA usually do not possess the same repertoire of virulence factors [i.e. staphylococcal enterotoxins (SEs), Panton-Valentine leukocidin (PVL)] and pathogenic properties (i.e. adhesion, internalization and immune evasion abilities) as human-adapted lineages (HA- and CA-MRSA) [3]. A phylogenomic study proposed that LA-MRSA ST398 evolved from a human methicillin-susceptible *S. aureus* (MSSA) clone which acquired resistance to both methicillin and tetracycline and lost the human-specific immune evasion gene cluster (IEC) after the jump from humans to livestock, attenuating its potential to cause zoonotic infection [13]. Based on evolutionary history and irrespective of methicillin-resistance, a formal definition of two major host-associated *S. aureus* ST398 clades has been proposed: the livestock clade (tetracycline resistant and IEC-negative) and the ancestral human clade (tetracycline susceptible and IEC-positive) [13, 14]. However, conventional definitions might be blurred in future due to the fast rate of MRSA evolution, i.e. changes in antibiotic susceptibility pattern and genetic signatures [2].

A high prevalence of multi-drug resistant (MDR) ST398 LA-MRSA has recently been documented by our group in a large cohort of healthy pigs farmed in southern Italy [15]. Given the potential exchange of MRSA between animals and humans, and the infection risk associated with human colonization by MDR LA-MRSA, the present study has been conducted to investigate: (*i*) the prevalence, genetic characteristics and antimicrobial resistance profile of MRSA isolated from swine farm workers in southern Italy, and (*ii*) the genome-based relatedness of human and animal MRSA isolates, for a better understanding of MRSA transmission to professionally-exposed farm workers.

## Methods

### Sampling

From January to March 2018, a cross-sectional prevalence study investigating *S. aureus* and MRSA carriage among 475 swine in 32 farms (25 with an intensive type of breeding and 7 with a non-intensive type) in the Calabria Region in southern Italy was conducted [15]. Farms were selected by both geographic distribution and convenience, mainly based on willingness to participate in the survey. Selected farms accounted for 8.99% of all swine farms from Calabria region (15,082 km^2^), and were located in all the five provinces: Catanzaro (CZ; 11 farms), Reggio Calabria (RC; 10 farms), Cosenza (CS; 5 farms), Vibo Valentia (VV; 3 farms), Crotone (KR; 3 farms). Among the 32 selected farms, 25 practiced intensive breeding, in which animals were in crowded conditions (i.e. animals confined to indoor fences), and 7 adopted non-intensive breeding systems (i.e. animals living in free-range conditions).

A total of 88 workers belonging to the previously selected swine farms, were sampled. All farm workers over the age of 18 who were present at the time of the visit were sampled, and all participants signed an informed consent form. The study was approved by the Ethical Committee of Azienda Ospedaliera Universitaria Policlinico “G. Martino”, Messina, Italy (decrete no.1158/2018).

On February 2019, resampling of workers was conducted, and 7 out of 88 previously sampled workers agreed to participate. In both samplings, a saline pre-moistened nasal swab was collected from both nostrils of each participating worker, and immediately transferred into 5 ml of high-salt enrichment broth [Mueller Hinton Broth (MHB) (Becton Dickinson) supplemented with 6.5% (w/vol) sodium chloride]. Tubes were incubated for 24 h at 37 °C.

### MRSA isolation and characterization

All samples were processed according to a previously described procedure for *S. aureus* and MRSA detection [15]. Briefly, aliquots of the enrichment broth (0.5 ml) were transferred to Phenol-Red Mannitol Broth (PRMB, 4.5 ml) (Becton Dickinson) and PRMB supplemented with 4 μg/ml of oxacillin (PRMB+OX, 4.5 ml). The two tubes were incubated for up to 48 h at 37 °C. If red-to-yellow colour change was observed in PRMB and PRMB+OX, 10-μl samples from PRMB+OX were plated on selective MRSA plates (Brilliance MRSA 2 agar, Oxoid). Suspected MRSA (blue) colonies were streaked on Muller Hinton Agar (MHA) (Becton Dickinson) supplemented with 4 μg/ml OX. If only the PRMB (without OX) turned yellow, presumptive *S. aureus* identification was obtained by the Staphytect plus test (Oxoid) on the bacterial pellet. The tubes that did not change colour after 48-h incubation at 37 °C were considered negative for the presence of both *S. aureus* and MRSA. All MRSA negative samples underwent a second screening procedure (look-back) to exclude the presence of MRSA in the first enrichment broth, as outlined previously [15].

Genomic DNA of MRSA isolates was extracted by the QIAamp DNA Mini Kit (QIAGEN) according to the manufacturer’s recommendations, except for the addition of 50 μg/ml lysostaphin (Sigma Aldridch) to improve staphylococcal cell lysis. A multiplex PCR with primers annealing to the 16S rDNA, *nuc* and *mecA* genes [16] was performed to confirm *S. aureus* identification and methicillin resistance.

MRSA isolates were characterized by *spa*, staphylococcal chromosomal cassette *mec* (SCC*mec*) and multi-locus sequence typing (MLST), as previously described [17–20].

The presence of *pvl* genes (*lukS*-*lukF*) coding for the PVL, *scn* and *tet(M)* was tested as previously described [14, 21]. The qPCR detection of enterotoxin-producing MRSA was performed using primers *SA-U* and *Sa3-r* [22]. The ST398-specific PCR was carried out with primer sets A07f/A07r [23]. Analysis of the A07 fragment was performed by amplicon sequencing with primers A07f/A07r.

Rep-MP3 and RAPD (Random Amplification of Polymorphic DNA) PCR were carried out as previously described using primer RW3A [24] and ERIC-2 [25], respectively. Fingerprints were digitally compared using the BioNumerics software (Version 6.6; Applied Maths). Cluster analysis with Dice similarity index (S_D_) based on the unweighted pair group method with arithmetic averages (UPGMA) was applied to generate dendrograms illustrating the relationships among fingerprints with the following comparison settings: optimization, 1.5%; minimum height, 0%; minimum surface, 0%; tolerance, 1%; tolerance change, 1%. An arbitrary cut-off value of 90% was chosen to assign rep-PCR clusters, named A to F. *S. aureus* ATCC 43300 was included as a control strain for analysis.

Antimicrobial susceptibility testing was performed by Vitek2 system (bioMérieux), using the AST-P588 card. All human MRSA isolates were tested using the same antibiotic panel as previously reported for swine isolates [15]. According to the CLSI interpretative criteria [26, 27], MRSA isolates were classified as susceptible, intermediate, or resistant. Strains classified as resistant and intermediate were included in the same group (non-susceptible).

### Whole genome sequencing (WGS), assembly and analysis

DNA libraries were prepared using Nextera XT v.3 (Illumina, San Diego, CA, USA) kit. WGS was performed using MiSeq (Illumina) platform with paired-end (2X 250-bp) operating mode. Fastq files of the paired-end reads were used as input for genome assemblies through the MEGAnnotator pipeline [28]. Multiple whole-genome alignments were performed and visualized using the Mauve progressive algorithm with default parameters [29].

Pairwise average nucleotide identity (ANI) was calculated with Jspecies v1.2.1 using the standard MUMmer algorithm [30]. Genome-wide single nucleotide polymorphism (SNP) analysis was performed using the CSI Phylogeny 1.4 server [31]. Sequences were aligned with the LA-ST398 MRSA reference strain S0385 genome (NC_017333, 2,872,582 nucleotides in size) for SNPs calls along 2,500,938 positions (87.1% of reference chromosome). A phylogenetic tree based by SNPs was visualized with MEGA (version X; ref. [32]).

### Data access

WGS data were submitted to the NCBI Sequence Read Archive (SRA) under BioProject PRJNA546229.

### Statistical analysis

Data analyses were performed using Sigma Plot software version 12.0 (Systat Software). Categorical variables were compared with the χ^2^ test or Fisher’s exact test when appropriate. Significance was defined as *P* ≤ 0.05.

## Results

### Prevalence of *S. aureus* and MRSA in farm workers

Eighty-eight workers from 32 pig farms voluntarily participated in the study. Mean age was 46.0 ± 13.5 (range: 21–90) and the majority (77/88; 87.5%) were men (Additional file 1: Table S1). In 25 of 32 farms, at least one worker tested positive for *S. aureus*, with an overall carriage rate of 55.7% (95% CI: 54.4–56.9%; 49/88 samples). In 11 farms, 19 non-duplicate MRSA isolates were identified from 88 sampled workers, with an MRSA carriage rate of 21.6% (95% CI: 21.1–22.1%). Of note, in 10 of 11 farms yielding MRSA isolates from workers, MRSA were also detected in animal samples.

Workers employed in farms with an intensive type of breeding had higher MRSA carriage rates compared with workers employed in farms that adopted a non-intensive breeding system (27.1% vs. 0%; Table 1). Higher colonization rates by *S. aureus* (irrespective of methicillin resistance) and MRSA were observed for workers reporting close contact with animals (65.8 and 26.0%, respectively), such as farm workers and veterinarians, compared with workers without animal contact (6.7 and 0%, respectively), such as household members or farm administration employees (Table 1). Interestingly, colonization by MRSA was significantly higher in workers aged < 50 compared to workers aged ≥50 (Table 1).

**Table 1:** Prevalence of *S. aureus* and MRSA in farm workers according to breeding type, animal contact and age

Variable	Category	No. of farms	No. of sampled workers	*S. aureus-*positive		MRSA-positive	
				No. (%)	*P*-value	No. (%)	*P*-value
Breeding type	Intensive	25	70	41 (58.6)	NS	19 (27.1)	0.01
	Non-intensive	7	18	8 (44.4)		0 (0)	
	Total	32	88	49 (55.7)		19 (21.6)	
Direct contact with swine	Yes	–	73	48 (65.8)	<0.0001	19 (26.0)	0.03
	No	–	15	1 (6.7)		0 (0)	
	Total		88	49 (55.7)		19 (21.6)	
Age	< 50	–	56	33 (58.9)	NS	17 (30.4)	0.01
	≥50	–	32	16 (50.0)		2 (6.25)	
	Total	–	88	49 (55.7)		19 (21.6)	

*NS* Not significant

### MLST, *spa* and SCC*mec* typing, toxinogenicity and antimicrobial susceptibility

All MRSA isolates from farm workers (*n* = 19) were assigned to ST398 by ST398-specific PCR [23] and MLST. Irrespective of their origin, all MRSA isolates belonged to the livestock-associated ST398 clade [14], defined as *tet(M)*-positive and *scn*-negative.

**Fig. 1:**
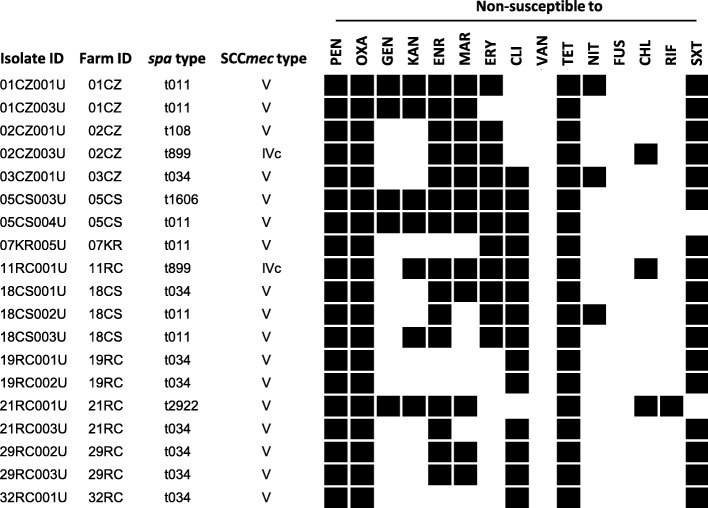
Epidemiological type and antibiotic resistance profile of 19 LA-MRSA ST398 isolates from swine farm workers. Black squares denote non-susceptibility (resistant or intermediate). PEN, penicillin; OXA, oxacillin; GEN, gentamycin; KAN, kanamycin; ENR, enrofloxacin; MAR, marbofloxacin; ERY, erythromycin; CLI, clindamycin; VAN; vancomycin; TET, tetracycline; NIT, nitrofurantoin; FUS, fusidic acid; CHL, chloramphenicol; RIF, rifampicin; SXT, trimethoprim-sulfamethoxazole. Non-susceptibility (resistant or intermediate) breakpoints (μg/ml) were: PEN≥0.25; OXA ≥ 4; GEN > 4; KAN > 16; ENR > 0.5; MAR > 1; ERY > 0.5; CLI > 0.5; VAN> 4; TET > 4; NIT > 32; FUS ≥ 4; CHL > 8; RIF > 1.0; SXT > 2/38

Six different *spa* types were detected, namely t034 (42.1%, 8 isolates), t011 (31.6%, 6 isolates), t899 (10.5%, 2 isolates), t1606, t108 and t2922 (1 isolate each, 5.3%). The majority of isolates harboured SCC*mec* type V (89.5% 17/19), whereas the remaining isolates were type IVc (10.5%, 2/19) (Fig. 1). As for the swine-associated MRSA [15], all MRSA isolates from workers were PVL- and SEs-negative (data not shown), and MDR (non-susceptible to at least three non β-lactams antimicrobial classes; Fig. 1), also showing comparable resistance frequencies (Additional file 2: Table S2).

### Inter- and intra-farm epidemiological correlations

**Fig. 2:**
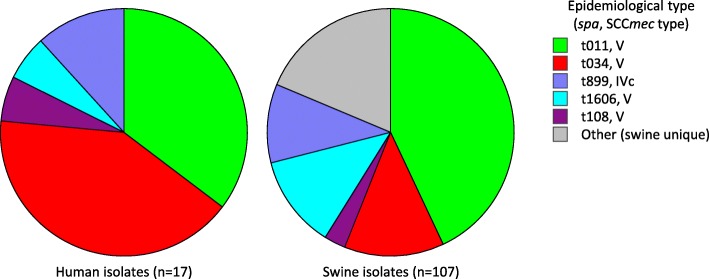
Combined *spa* and SCC*mec* types of ST398 MRSA isolates from farm workers and swine

**Fig. 3:**
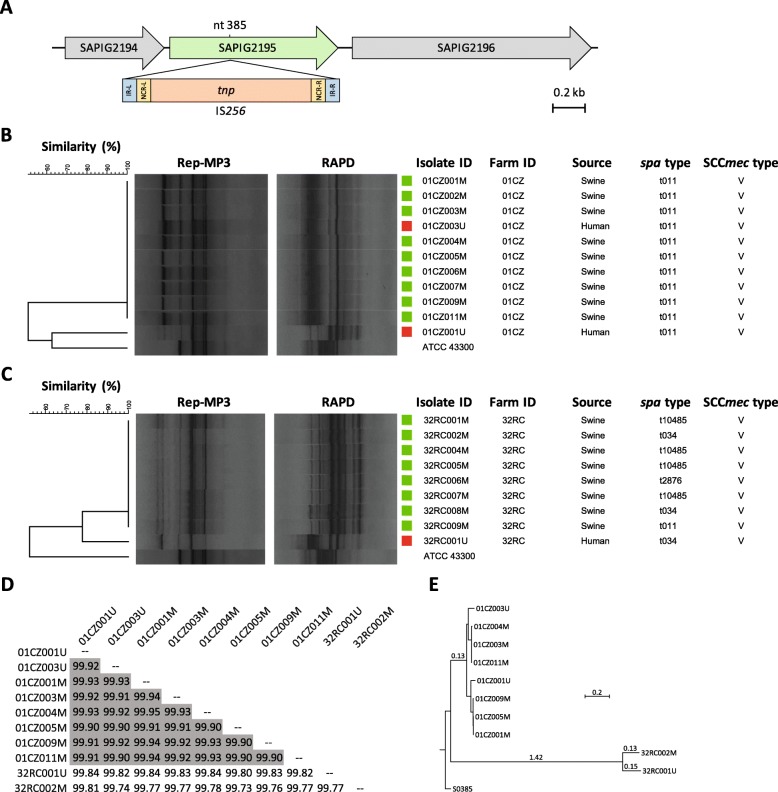
Relevant genetic features of ST398 LA-MRSA from swine and farm workers. **a** IS*256* insertion in the SAPIG2195 gene, detected in all t011 MRSA isolates from farm ID 05CS. The insertion position (nt 385) is relative to the SAPIG2195 coding sequence of *S. aureus* WCH-SK2 (CP031537). **b**, **c** Rep-MP3 PCR and RAPD fingerprints of MRSA isolates from workers and swine belonging to farm ID 01CZ and 32RC, respectively. The dendrogram was generated with BioNumerics using UPGMA and the Dice coefficient. *S. aureus* ATCC 43300 was included as outgroup for analysis. Squares denote the source of MRSA; red, human; green, swine. **d** Pairwise ANI comparison matrix of MRSA genomes, calculated with JSpecies. ANI values > 99.90% are in grey. **e** Phylogenetic tree based on SNPs analysis obtained by CSI phylogeny and visualized with MEGA. LA-MRSA strain S0385 was used as reference. Branch lengths are the number of substitutions per site

A similar distribution of epidemiological types (*spa* and SCC*mec* combination) was observed for human (*n* = 17) and swine (*n* = 107) MRSA isolates in the ten farms from which MRSA had been isolated from both swine [15] and workers (Fig. 2). All but one (16/17) MRSA isolates from workers showed the same *spa* and SCC*mec* type combination as at least one swine-associated MRSA isolate from the same farm. Remarkably, the t011 MRSA isolate from an employee of farm ID 05CS (isolate ID 05CS004U) (Additional file 1: Table S1) carried the typical IS*256* insertion signature in the A07 fragment of the SAPIG2195 gene (A07::IS*256*; Fig. 3a), which was previously documented in all t011 swine-associated MRSA isolates from the same farm [15].

To substantiate the genetic identity between human and animal MRSA showing the same *spa* and SCC*mec* type, isolates from workers and swine were also analysed by rep-PCR and RAPD fingerprinting. Six different rep-PCR clusters (S_D_ > 90%), designated A through F, were defined (Additional file 3: Figure S1). Excluding one human isolate (18CS001U) for which no animal correlate was detected due to different *spa* and SCC*mec* types relative to the swine counterpart, 14 out of 16 MRSA belonged to the same rep-PCR cluster of at least one swine-related MRSA of the same farm (Additional file 3: Figure S1). The remaining 2 isolates (from farms ID 01CZ and 32RC) were not identical to the swine-associated MRSA from the same farm. Albeit showing the same *spa* and SCC*mec* type, these two isolates slightly differed in rep-PCR fingerprints compared with porcine MRSA isolates of the same farm (Table 2). Minor differences in the genetic background of these isolates were also inferred from RAPD fingerprinting (Fig. 3b and c). Among human isolates (*n* = 19), the most prevalent types were t011, V, C (4 isolates from 3 farms; 21.0%) and t034, V, A (3 isolates from 2 farms; 15.8%) (Table 2).

**Table 2:** Epidemiological correlations between MRSA isolates from farm workers and swine

Farm ID	Human		Swine	
	No. MRSA-positive/No. of sampled workers	Combined type^a^ (No. of isolates)	No. MRSA-positive/No. of sampled swine	Combined type^a^ (No. of isolates)
01CZ	2/5	**t011, V, A (1)**	9/12	**t011, V, A (9)**
		t011, V, C (1)^b^		
02CZ	2/3	**t108, V, C (1)**	8/10	**t108, V, C (3)**
		**t899, IVc, C (1)**		**t899, IVc, C (1)**
				t034, V, C (4)
03CZ	1/2	**t034, V, E (1)**	5/10	**t034, V, E (2)**
				t571, V, D (2)
				t034, V, A (1)
05CS	2/5	**t011**^**c**^**, V, C (1)**	24/30	**t011**^**c**^**, V, C (2)**
		**t1606, V, C (1)**		**t1606, V, C (13)**
				t011^c^, V, A (8)
				t5524, V, C (1)
07KR^d^	1/5	**t011, V, C (1)**	18/19	**t011, V, C (11)**
				t011, V, A (7)
11RC	1/2	**t899, IVc, F (1)**	10/13	**t899, IVc, F (10)**
18CS^d^	3/3	**t011, V, C (2)**	10/10	**t011, V, C (8)**
		t034, V, C (1)		t1184, V, C (2)
19RC	2/2	**t034, V, B (2)**	6/20	**t034, V, B (1)**
				t034, V, D (2)
				t1793, V, B (2)
				t571, V, D (1)
21RC	2/4	t034, V, D (1)	0/16	–
		t2922, V, D (1)		–
29RC	2/3	**t034, V, A (2)**	9/20	**t034, V, A (2)**
				t899, V, C (7)
32RC	1/2	t034, V, A (1)^b^	8/9	t034, V, D (2)
				t10485, V, D (4)
				t011, V, D (1)
				t2876, V, D (1)

Human and swine isolates for which an epidemiological association has been identified are highlighted in bold

^a^*spa*, SCC*mec*, Rep-MP3 type

^b^For these isolates results have also been confirmed by RAPD typing

^c^t011 isolate(s) displaying the IS*256* insertion signature in the A07 fragment of the SAPIG2195 coding region (IS*256*::A07) (ref. [15])

^d^Trading of animals during the survey period has been documented between farm ID 07KR (seller) and 18CS (purchaser) (ref. [15])

To address the differences in fingerprinting results, 8 isolates from farm ID 01CZ and 2 isolates from farm 32RC were analysed by WGS, and their relatedness was assessed by ANI, SNPs analysis and whole-genome alignments. All human and swine isolates from farm ID 01CZ were nearly indistinguishable at the genome level, showing > 99.90% ANI in pairwise comparisons [33] (Fig. 3d). Conversely, isolates from farm ID 32RC displayed 99.77% ANI (Fig. 3d), resulting highly related but not identical. SNPs analysis confirmed these results (Fig. 3e), since isolates from farm ID 01CZ differed by 27.9 ± 15.9 SNPs (range 0–49), whereas isolates from farm ID 32RC differed by 91 SNPs.

**Fig. 4:**
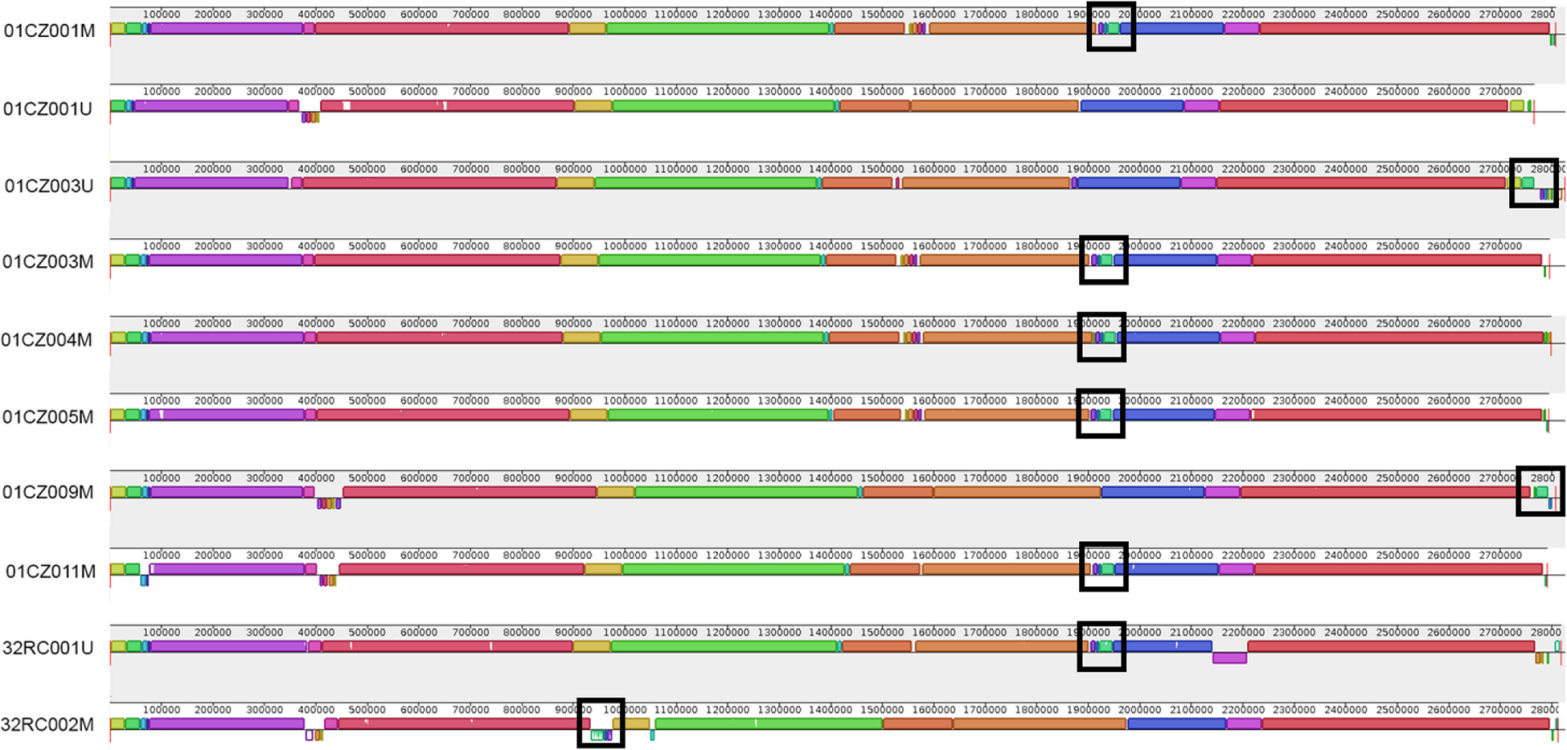
Multiple whole-genome alignment of MRSA from farms 01CZ and 32RC. Fully assembled sequences were compared with ProgressiveMauve using default parameters. The figure was generated by MAUVE viewer. Boxes with identical colours represent local colinear blocks (LCB), i.e. homologous DNA regions without sequence rearrangements shared by two or more genomes. LCBs reported below the horizontal axis represent reverse complements of the arbitrary chosen reference LCB (01CZ001M). White-filled portions denote areas of low similarity. Boxes define the ca. 43-kb region that was absent in 01CZ001U and partly conserved in 32RC002M

Multiple whole-genome alignments highlighted a similar genome structure for all strains, including a ~ 43-kb region which was conserved in all the isolates except 01CZ001U and 32RC002M (Fig. 4). This region was 99% identical to the *Staphylococcus* phage Sebago (43,878 kb, genome ID: MK618716.1), and its variation in 01CZ001U and 32RC002M could account for the observed differences in rep-PCR and RAPD fingerprints. Thus, in farms providing both swine and human MRSA, genotyping results indicate that 94.1% (16/17) of isolates from workers were identical or closely related to at least one isolate from swine of the same farm.

### Follow-up investigation

Out of 88 workers who participated in the survey, 7 agreed to be resampled 1 year after the first screening. Four workers who were MRSA-negative at the first sampling were confirmed negative, whereas two of three workers who were MRSA-positive at the first sampling were confirmed positive. Interestingly, these two workers carried an identical MRSA strain as the primary isolate, i.e. belonging to the livestock ST398 clade and showing identical *spa*, SCC*mec*, rep-PCR and RAPD types (Additional file 4: Figure S2).

## Discussion

LA-MRSA ST398 is an occupational hazard for people in direct contact with livestock animals. Indeed, swine are a major reservoir of this staphylococcal lineage in western countries, including Italy, where a high prevalence of ST398 has been documented in swine farms over the past decade [9, 15, 34]. Worryingly, an increasing number of LA-MRSA ST398 infections has been reported in professionally exposed workers or in people living nearby high-density swine farming areas [3–5]. The clinical spectrum of LA-MRSA ST398 infection varies from SSTIs to invasive infections, including bloodstream infections, pneumonia and bone and joint infections (reviewed by ref. [3]). Of note, LA-MRSA ST398 has previously been reported to cause pelvic multiloculated abscess and severe necrotizing fasciitis in two Italian farm workers [11, 12].

To gain insight of the risk associated with professional exposure to MRSA-colonized pigs and trace the epidemiological trajectories of MRSA in pig farming, we investigated the MRSA prevalence in workers of swine farms which had concomitantly been screened for MRSA colonization of farmed pigs [15].

A high rate (55.7%) of *S. aureus* nasal carriage was observed in farm workers, with an MRSA prevalence of 21.6%. Although high MRSA colonization rates have been documented for swine farmers in different European countries [7, 8, 10], here we report higher rates of MRSA nasal colonization in swine farm workers from southern Italy, compared with previous surveys from the same region (7.3–8%; refs [9, 35]).

MRSA colonization was significantly higher in workers reporting direct contact with swine (farm workers and veterinarians), compared with other farm employees, indicating that the carrier status is associated with direct animal contact. In agreement with a previous report, MRSA colonization was age-related [8], and workers aged <50 years had a higher chance of being MRSA nasal carriers, compared with their older peers. Remarkably, farms which had adopted a non-intensive breeding system showed lower colonization rates with *S. aureus* and never yielded MRSA, in line with previous studies [7, 36]. Although limited in sample size, follow-up screening provided evidence of LA-MRSA persistence or re-colonization in some workers, given that carriage of the same MRSA strain was demonstrated 1 year after primary sampling. This strengthens the notion that frequent animal contact, especially in intensive swine breeding, is a major risk factors for persistent colonization with LA-MRSA in farm workers [36, 37]. In our previous survey, a trade of pigs between farm ID 07KR (seller) and farm ID 18CS (purchaser) has been documented [15], and MRSA isolates with an identical epidemiological type (t011, V, C) were detected in the majority of animals from these two farms. Intriguingly, workers of these farms were colonized by the same MRSA strain detected in animals (see Table 2, and Additional file 3: Figure S1), and two of them were found to be (re)colonized by the same strain (ID 07KR005U and 18CS002U) upon follow-up screening (Additional file 4: Figure S2). This observation suggests that inter-farm pig movements drive the spread of MRSA ST398 clones, leading to an increased risk of MRSA transmission to workers [38]. Therefore, periodical screening of LA-MRSA carriage in farm workers and animals should be implemented to reduce the inter-farm spreading of LA-MRSA.

Irrespective of the source (either workers or swine), all ST398 MRSA isolates analysed in this survey belong to the livestock clade, being *tet(M)-*positive and *scn*-negative, and displayed epidemiological profiles (i.e. *spa* and SCC*mec* type) mirroring the diversity of LA-MRSA ST398 so far reported in Europe [7, 10]. Moreover, both human and swine MRSA isolates were MDR, and showed similar resistance profiles, especially to drugs commonly used in pig husbandry (tetracyclines, lincosamides, macrolides and fluoroquinolones). Nonetheless, all isolates were susceptible to vancomycin and all but one to rifampicin, and were negative for PVL and ETs production, consistent with the attenuated virulence potential reported for LA-MRSA ST398 [3]. However, the possibility that LA-MRSA can evolve towards a more virulent pathotype should not be disregarded, as many staphylococcal virulence genes reside on mobile genetic elements [39] and it has recently been demonstrated that lysogenization of LA-MRSA CC398 strains by virulence-associated phages leads to the production of new virulence factors [40].

By combining epidemiological typing (*spa*, SCC*mec*, MLST) with DNA fingerprinting (Rep- and RAPD PCR) and whole genome analysis results, identity or close relatedness between human and swine MRSA isolates from the same farm was demonstrated. Our study highlights the power of WGS in epidemiological investigations, since apparent difference in DNA fingerprints could be explained by insertion of the *Staphylococcus* phage Sebago within identical genome scaffolds. Thus, typing data suggest unidirectional transmission of LA-MRSA from pigs to workers, either by direct animal contact or indirectly, through the farm environment, given that LA-MRSA can survive in dust for weeks [41].

## Conclusion

The results of this study highlight the high prevalence of ST398 LA-MRSA in pig farm workers from southern Italy, and associates the risk of MRSA colonization with intensive farming and direct animal contact. Our findings should raise the awareness of the risk of transmission of MDR LA-MRSA ST398 from animal to exposed workers. The public health and veterinary importance of MDR LA-MRSA highlights the need for effective interventions to control the spreading of this zoonotic lineage among livestock and in the community. Periodic screening of animals and farm workers and, eventually, decolonization measures could lower the risk of intra- and inter-farm MRSA transmission. In the One Health perspective, guidelines and recommendations aimed at preventing MDR LA-MRSA transmission through the food chain and reducing antimicrobial consumption in intensive animal husbandry are advocated.

## Supplementary information

**Additional file 1: Table S1.** Characteristics of swine farm workers.

**Additional file 2: Table S2.** Resistance to individual antimicrobials in MRSA isolates obtained from 19 farm workers and 107 pigs.

**Additional file 3: Figure S1.** Rep-MP3 PCR analysis of MRSA isolates from workers (*n* = 19) and swine (*n* = 107).

**Additional file 4: Figure S2.** Rep-MP3 PCR and RAPD fingerprints of MRSA isolates obtained from the follow-up investigation of farm workers.

## Data Availability

All data generated or analysed during this study are included in this published article and its supplementary information files.

## Abbreviations

ANI: average nucleotide identity
CA-MRSA: community-associated methicillin-resistant *Staphylococcus aureus*
HA-MRSA: hospital-associated methicillin-resistant *Staphylococcus aureus*
IEC: immune evasion gene cluster
LA-MRSA: livestock-associated methicillin-resistant *Staphylococcus aureus*
MDR: multi-drug resistant
MLST: multi-locus sequence typing
MRSA: methicillin-resistant *Staphylococcus aureus*
PVL: Panton-Valentine leucocidin
RAPD: random amplification of polymorphic DNA
SCC*mec*: staphylococcal chromosomal cassette *mec*
SE: staphylococcal enterotoxin
ST: sequence type
UPGMA: unweighted pair group method with arithmetic averages
WGS: whole genome sequencing
