# Supplementary material for: Unidirectional animal-to-human transmission of methicillin-resistant Staphylococcus aureus ST398 in pig farming; evidence from a surveillance study in southern Italy
Source: Antimicrob Resist Infect Control. 2019 Nov 21;8:187. doi: 10.1186/s13756-019-0650-z (PMC6873530; doi:10.1186/s13756-019-0650-z)
Supplement: Supplementary file 1 — Additional file 1: Table S1. Characteristics of swine farm workers. [file 13756_2019_650_MOESM1_ESM.pdf]

Table S1. Characteristics of swine farm workers

Sample ID	Farm ID	Sex	Age	Nationality	Employement	Direct contact with swine	*S. aureus* nasal carriage	MRSA nasal carriage
01CZ001U	01CZ	M	55	Italy	Farm worker	Yes	Yes	Yes
01CZ002U	01CZ	M	35	Côte d'Ivoire	Farm worker	Yes	Yes	No
01CZ003U	01CZ	M	49	Italy	Farm worker	Yes	Yes	Yes
01CZ004U	01CZ	M	40	Italy	Other[a]	No	No	No
01CZ005U	01CZ	M	84	Italy	Other[a]	No	No	No
02CZ001U	02CZ	M	39	Italy	Farm worker	Yes	Yes	Yes
02CZ002U	02CZ	F	36	Italy	Other[a]	No	No	No
02CZ003U	02CZ	M	39	Romania	Farm worker	Yes	Yes	Yes
03CZ001U	03CZ	M	31	Italy	Farm worker	Yes	Yes	Yes
03CZ002U	03CZ	M	48	Italy	Farm worker	Yes	No	No
04CZ001U	04CZ	F	79	Italy	Other[a]	No	No	No
04CZ002U	04CZ	F	53	Italy	Farm worker	Yes	Yes	No
04CZ003U	04CZ	M	51	Italy	Farm worker	Yes	Yes	No
04CZ004U	04CZ	M	21	Italy	Farm worker	Yes	Yes	No
04CZ005U	04CZ	M	49	Italy	Farm worker	Yes	Yes	No
04CZ006U	04CZ	M	45	Italy	Farm worker	Yes	No	No
05CS001U	05CS	M	54	Italy	Farm worker	Yes	Yes	No
05CS002U	05CS	M	57	Italy	Farm worker	Yes	No	No
05CS003U	05CS	M	46	Italy	Farm worker	Yes	Yes	Yes
05CS004U	05CS	M	47	Italy	Farm worker	Yes	Yes	Yes
05CS005U	05CS	M	46	Italy	Farm worker	Yes	Yes	No
06CS001U	06CS	M	58	Italy	Veterinarian	Yes	No	No
06CS002U	06CS	M	46	Italy	Farm worker	Yes	Yes	No
07KR001U	07KR	M	51	Italy	Farm worker	Yes	No	No
07KR002U	07KR	M	40	Italy	Farm worker	Yes	No	No
07KR003U	07KR	M	21	Italy	Farm worker	Yes	Yes	No
07KR004U	07KR	M	21	Italy	Farm worker	Yes	Yes	No
07KR005U	07KR	M	49	Italy	Veterinarian	Yes	Yes	Yes
08CZ001U	08CZ	M	49	Italy	Farm worker	Yes	No	No
08CZ002U	08CZ	M	44	Italy	Other[a]	No	Yes	No
08CZ003U	08CZ	M	43	Italy	Other[a]	No	No	No
08CZ004U	08CZ	M	33	Italy	Farm worker	Yes	No	No
08CZ005U	08CZ	M	57	Italy	Farm worker	Yes	Yes	No
08CZ006U	08CZ	M	45	Italy	Farm worker	Yes	Yes	No
09CZ001U	09CZ	M	61	Italy	Farm worker	Yes	Yes	No
10RC001U	10RC	M	57	India	Farm worker	Yes	Yes	No
10RC002U	10RC	M	66	Italy	Other[a]	No	No	No
11RC001U	11RC	M	26	Italy	Farm worker	Yes	Yes	Yes
11RC002U	11RC	M	62	Italy	Farm worker	Yes	No	No
12RC001U	12RC	M	50	Italy	Farm worker	Yes	Yes	No
12RC002U	12RC	M	34	Italy	Other[a]	No	No	No
13CZ001U	13CZ	M	58	Italy	Farm worker	Yes	No	No
13CZ002U	13CZ	F	57	Italy	Other[a]	No	No	No
13CZ003U	13CZ	F	28	Italy	Other[a]	No	No	No
14CZ001U	14CZ	M	61	Italy	Farm worker	Yes	No	No
14CZ002U	14CZ	F	62	Italy	Farm worker	Yes	No	No

15CZ001U	15CZ	F	33	Italy	Farm worker	Yes	No	No
15CZ002U	15CZ	M	44	Italy	Farm worker	Yes	No	No
16CS001U	16CS	M	42	Italy	Farm worker	Yes	Yes	No
16CS002U	16CS	M	46	Romania	Farm worker	Yes	Yes	No
16CS003U	16CS	M	42	Italy	Veterinarian	Yes	No	No
16CS004U	16CS	M	61	Italy	Veterinarian	Yes	No	No
17CS001U	17CS	M	60	Italy	Farm worker	Yes	Yes	No
17CS002U	17CS	F	40	Italy	Other[a]	No	No	No
17CS003U	17CS	M	59	Albania	Farm worker	Yes	Yes	No
18CS001U	18CS	M	38	Italy	Farm worker	Yes	Yes	Yes
18CS002U	18CS	M	49	Italy	Farm worker	Yes	Yes	Yes
18CS003U	18CS	M	32	Italy	Farm worker	Yes	Yes	Yes
19RC001U	19RC	M	38	Italy	Farm worker	Yes	Yes	Yes
19RC002U	19RC	M	33	Italy	Farm worker	Yes	Yes	Yes
20RC001U	20RC	M	33	Italy	Other[a]	No	No	No
20RC002U	20RC	M	41	Italy	Other[a]	No	No	No
20RC003U	20RC	M	40	India	Farm worker	Yes	Yes	No
20RC004U	20RC	M	59	Italy	Farm worker	Yes	No	No
21RC001U	21RC	M	45	India	Farm worker	Yes	Yes	Yes
21RC002U	21RC	M	90	Italy	Other[a]	No	No	No
21RC003U	21RC	M	61	Italy	Veterinarian	Yes	Yes	Yes
21RC004U	21RC	M	60	Italy	Veterinarian	Yes	Yes	No
22CZ001U	22CZ	M	24	Italy	Farm worker	Yes	No	No
23CZ001U	23CZ	M	46	Switzerland	Farm worker	Yes	No	No
24KR001U	24KR	M	24	Italy	Farm worker	Yes	Yes	No
24KR002U	24KR	M	51	Italy	Farm worker	Yes	Yes	No
24KR003U	24KR	M	41	Italy	Farm worker	Yes	No	No
25KR001U	25KR	M	50	Romania	Farm worker	Yes	Yes	No
25KR002U	25KR	M	64	Italy	Veterinarian	Yes	No	No
26VV001U	26VV	M	38	Romania	Farm worker	Yes	No	No
26VV002U	26VV	M	31	Italy	Farm worker	Yes	No	No
27VV001U	27VV	M	52	Italy	Other[a]	No	No	No
27VV002U	27VV	M	41	Italy	Farm worker	Yes	No	No
28VV001U	28VV	M	47	Italy	Farm worker	Yes	Yes	No
29RC001U	29RC	F	31	Italy	Farm worker	Yes	Yes	No
29RC002U	29RC	M	35	Italy	Farm worker	Yes	Yes	Yes
29RC003U	29RC	F	33	Italy	Farm worker	Yes	Yes	Yes
30RC001U	30RC	F	58	Italy	Farm worker	Yes	Yes	No
31RC001U	31RC	M	25	Italy	Farm worker	Yes	Yes	No
31RC002U	31RC	M	58	Italy	Veterinarian	Yes	Yes	No
32RC001U	32RC	M	32	Ukraine	Farm worker	Yes	Yes	Yes
32RC002U	32RC	M	37	Italy	Farm worker	Yes	No	No

[a]Household member or external farm employee
