# Supplementary material for: Unidirectional animal-to-human transmission of methicillin-resistant Staphylococcus aureus ST398 in pig farming; evidence from a surveillance study in southern Italy
Source: Antimicrob Resist Infect Control. 2019 Nov 21;8:187. doi: 10.1186/s13756-019-0650-z (PMC6873530; doi:10.1186/s13756-019-0650-z)
Supplement: Supplementary file 2 — Additional file 2: Table S2. Resistance to individual antimicrobials in MRSA isolates obtained from 19 farm workers and 107 pigs. [file 13756_2019_650_MOESM2_ESM.pdf]

Table S2. Resistance to individual antimicrobials in MRSA isolates obtained from 19 farm workers and 107 pigs

Antimicrobial target	Antimicrobial class	Antimicrobial[a]	No. of non-susceptible (R+I) isolates (%)[b]	
			Farm workers	Swine
Peptidoglycan synthesis				
	β-lactams	PEN	19 (100)	107 (100)
		OXA	19 (100)	107 (100)
	Glycopeptides	VAN	0 (0)	0 (0)
DNA synthesis				
	Fluoroquinolones	ENR	15 (78.9)	76 (71.0)
		MAR	12 (63.2)	59 (55.1)
Protein synthesis				
	Aminoglycosides	GEN	5 (26.3)	42 (39.3)
		KAN	7 (36.8)	44 (41.1)
	Macrolides	ERY	11 (57.9)	70 (65.4)
	Lincosamides	CLI	14 (73.7)	96 (89.7)
	Tetracyclines	TET	19 (100)	107 (100)
	Fucidanes	FUS	0 (0)	0 (0)
	Phenicols	CHL	3 (15.8)	12 (11.2)
	Ansamycins	RIF	1 (5.3)	1 (0.9)
Others				
	Nitrofuranes	NIT	3 (15.8)	4 (3.7)
	Folate pathways inhibitors	SXT	17 (89.5)	74 (69.2)

[a] PEN, penicillin; OXA, oxacillin; VAN; vancomicin; ENR, enrofloxacin; MAR, marbofloxacin; GEN, gentamycin; KAN, kanamycin; ERY, erythromycin; CLI, clindamycin; TET, tetracycline; FUS, fusidic acid; CHL, chloramphenicol; RIF, rifampicin; NIT, nitrofurantoin; SXT, trimethoprim-sulfamethoxazole.

[b] Isolates showing resistance (R) and intermediate susceptibility (I) were classified as non-susceptible.
