# Supplementary material for: Unidirectional animal-to-human transmission of methicillin-resistant Staphylococcus aureus ST398 in pig farming; evidence from a surveillance study in southern Italy
Source: Antimicrob Resist Infect Control. 2019 Nov 21;8:187. doi: 10.1186/s13756-019-0650-z (PMC6873530; doi:10.1186/s13756-019-0650-z)
Supplement: Supplementary file 3 — Additional file 3: Figure S1. Rep-MP3 PCR analysis of MRSA isolates from workers (n = 19) and swine (n = 107). [file 13756_2019_650_MOESM3_ESM.pdf]

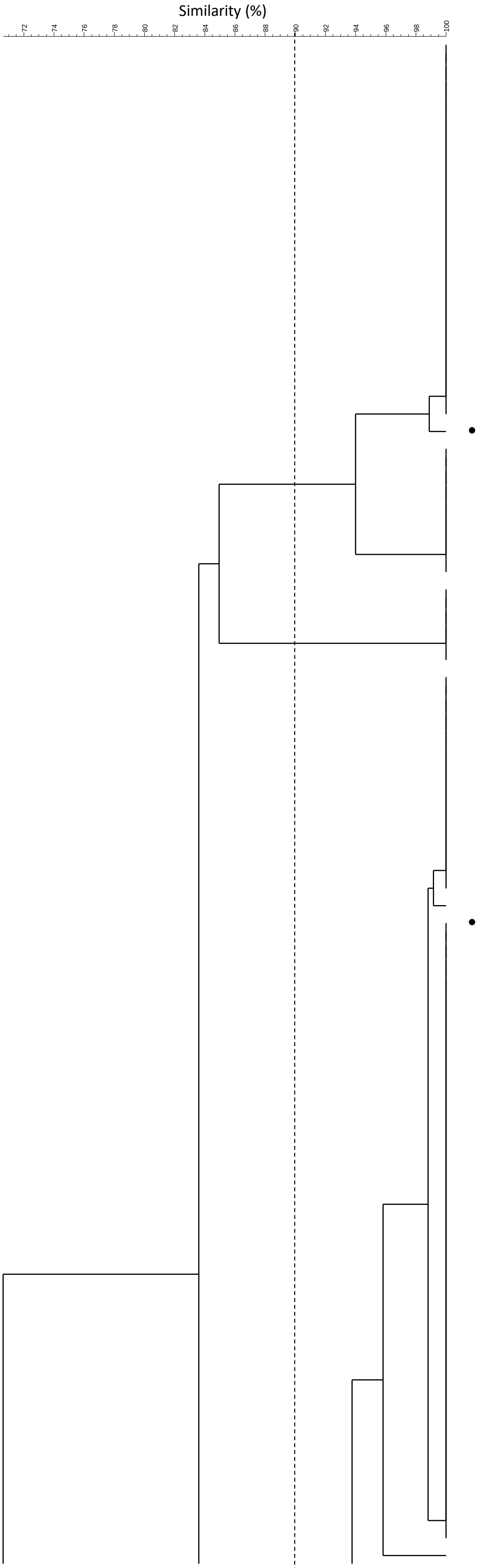

Isolate ID	Farm ID	Source	*spa* type	SCC*mec* type	Rep-PCRcluster
01CZ001M	01CZ	Swine	t011	V	
01CZ002M	01CZ	Swine	t011	V	
01CZ003M	01CZ	Swine	t011	V	
01CZ003U	01CZ	Human	t011	V	
01CZ004M	01CZ	Swine	t011	V	
01CZ005M	01CZ	Swine	t011	V	
01CZ006M	01CZ	Swine	t011	V	
01CZ007M	01CZ	Swine	t011	V	
01CZ009M	01CZ	Swine	t011	V	
01CZ011M	01CZ	Swine	t011	V	
03CZ010M	03CZ	Swine	t034	V	
07KR010M	07KR	Swine	t011	V	
07KR012M	07KR	Swine	t011	V	
07KR013M	07KR	Swine	t011	V	
07KR014M	07KR	Swine	t011	V	
07KR015M	07KR	Swine	t011	V	A
07KR018M	07KR	Swine	t011	V	
07KR019M	07KR	Swine	t011	V	
29RC002U	29RC	Human	t034	V	
29RC003U	29RC	Human	t034	V	
29RC009M	29RC	Swine	t034	V	
29RC010M	29RC	Swine	t034	V	
32RC001U	32RC	Human	t034	V	
05CS001M	05CS	Swine	t011	V	
05CS008M	05CS	Swine	t011	V	
05CS009M	05CS	Swine	t011	V	
05CS010M	05CS	Swine	t011	V	
05CS012M	05CS	Swine	t011	V	
05CS013M	05CS	Swine	t011	V	
05CS019M	05CS	Swine	t011	V	
05CS020M	05CS	Swine	t011	V	
19RC001U	19RC	Human	t034	V	
19RC002U	19RC	Human	t034	V	
19RC007M	19RC	Swine	t1793	V	B
19RC013M	19RC	Swine	t034	V	
19RC017M	19RC	Swine	t1793	V	
05CS011M	05CS	Swine	t1606	V	
18CS010M	18CS	Swine	t011	V	
05CS027M	05CS	Swine	t1606	V	
07KR001M	07KR	Swine	t011	V	
07KR002M	07KR	Swine	t011	V	
07KR003M	07KR	Swine	t011	V	
07KR005U	07KR	Human	t011	V	
07KR006M	07KR	Swine	t011	V	
18CS001M	18CS	Swine	t1184	V	
18CS002M	18CS	Swine	t011	V	
18CS004M	18CS	Swine	t011	V	
18CS005M	18CS	Swine	t011	V	
18CS008M	18CS	Swine	t011	V	
18CS006M	18CS	Swine	t1184	V	
01CZ001U	01CZ	Human	t011	V	
02CZ001M	02CZ	Swine	t108	V	
02CZ001U	02CZ	Human	t108	V	
02CZ002M	02CZ	Swine	t108	V	
02CZ003M	02CZ	Swine	t034	V	
02CZ003U	02CZ	Human	t899	IVc	
02CZ005M	02CZ	Swine	t034	V	
02CZ006M	02CZ	Swine	t034	V	
02CZ007M	02CZ	Swine	t034	V	
02CZ008M	02CZ	Swine	t108	V	
02CZ010M	02CZ	Swine	t899	IVc	
05CS002M	05CS	Swine	t1606	V	
05CS003M	05CS	Swine	t1606	V	C
05CS003U	05CS	Human	t1606	V	
05CS004M	05CS	Swine	t5524	V	
05CS004U	05CS	Human	t011	V	
05CS006M	05CS	Swine	t1606	V	
05CS014M	05CS	Swine	t1606	V	
05CS015M	05CS	Swine	t1606	V	
05CS016M	05CS	Swine	t011	V	
05CS021M	05CS	Swine	t1606	V	
05CS023M	05CS	Swine	t1606	V	
05CS024M	05CS	Swine	t011	V	
05CS025M	05CS	Swine	t1606	V	
05CS026M	05CS	Swine	t1606	V	
05CS029M	05CS	Swine	t1606	V	
05CS030M	05CS	Swine	t1606	V	
07KR004M	07KR	Swine	t011	V	
07KR007M	07KR	Swine	t011	V	
07KR008M	07KR	Swine	t011	V	
07KR009M	07KR	Swine	t011	V	
07KR011M	07KR	Swine	t011	V	
07KR016M	07KR	Swine	t011	V	
18CS001U	18CS	Human	t034	V	
18CS002U	18CS	Human	t011	V	
18CS003U	18CS	Human	t011	V	
07KR017M	07KR	Swine	t011	V	

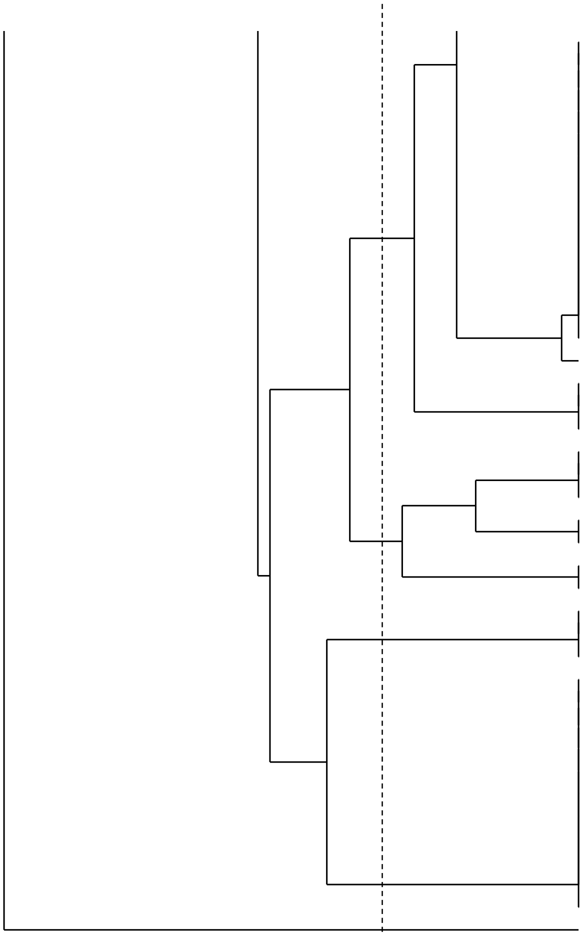

Isolate ID	Farm ID	Source	*spa* type	SCC*mec* type	Rep-PCRcluster
29RC001M	29RC	Swine	t899	V	
29RC002M	29RC	Swine	t899	V	
29RC003M	29RC	Swine	t899	V	
29RC004M	29RC	Swine	t899	V	
29RC007M	29RC	Swine	t899	V	
29RC011M	29RC	Swine	t899	V	
29RC014M	29RC	Swine	t899	V	
32RC001M	32RC	Swine	t10485	V	
32RC002M	32RC	Swine	t034	V	C
32RC005M	32RC	Swine	t10485	V	
32RC006M	32RC	Swine	t2876	V	
32RC007M	32RC	Swine	t10485	V	
32RC008M	32RC	Swine	t034	V	
32RC009M	32RC	Swine	t011	V	
32RC004M	32RC	Swine	t10485	V	
18CS003M	18CS	Swine	t011	V	
18CS007M	18CS	Swine	t011	V	
18CS009M	18CS	Swine	t011	V	
19RC006M	19RC	Swine	t571	V	
19RC019M	19RC	Swine	t034	V	
19RC020M	19RC	Swine	t034	V	
03CZ006M	03CZ	Swine	t571	V	D
03CZ009M	03CZ	Swine	t571	V	
21RC001U	21RC	Human	t2922	V	
21RC003U	21RC	Human	t034	V	
03CZ004M	03CZ	Swine	t034	V	
03CZ001U	03CZ	Human	t034	V	E
03CZ007M	03CZ	Swine	t034	V	
11RC001M	11RC	Swine	t899	IVc	
11RC001U	11RC	Human	t899	IVc	
11RC002M	11RC	Swine	t899	IVc	
11RC003M	11RC	Swine	t899	IVc	
11RC004M	11RC	Swine	t899	IVc	
11RC005M	11RC	Swine	t899	IVc	F
11RC006M	11RC	Swine	t899	IVc	
11RC007M	11RC	Swine	t899	IVc	
11RC008M	11RC	Swine	t899	IVc	
11RC010M	11RC	Swine	t899	IVc	
11RC011M	11RC	Swine	t899	IVc	
ATCC 43300					G

Figure S1. Rep-MP3 PCR analysis of MRSA isolates from workers (n=19) and swine (n=107). The dendrogram was generated with BioNumerics (Applied Maths) using the unweighted pair-group method with arithmetic averages (UPGMA) and the Dice coefficient. Boxes (A to F) denote clusters of isolates with a similar profile (>90% similarity). Human MRSA isolates (2/19) for which a correlation with swine isolates from the same farm has not been observed are marked with a black circle. *S. aureus* ATCC 43300 was included as outgroup for analysis.
