# Supplementary material for: Unidirectional animal-to-human transmission of methicillin-resistant Staphylococcus aureus ST398 in pig farming; evidence from a surveillance study in southern Italy
Source: Antimicrob Resist Infect Control. 2019 Nov 21;8:187. doi: 10.1186/s13756-019-0650-z (PMC6873530; doi:10.1186/s13756-019-0650-z)
Supplement: Supplementary file 4 — Additional file 4: Figure S2. Rep-MP3 PCR and RAPD fingerprints of MRSA isolates obtained from the follow-up investigation of farm workers. [file 13756_2019_650_MOESM4_ESM.pdf]

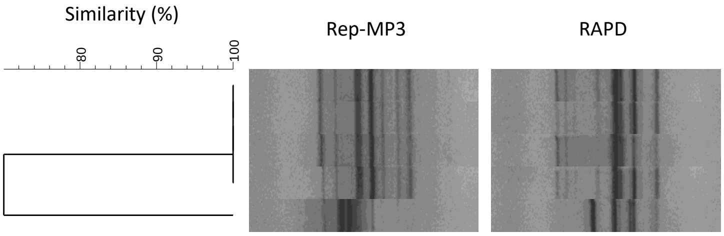

Isolate ID	Investigation	*spa* type	SCC*mec* type
07KR005U	Primary screening	t011	V
07KR005U-F	Follow-up	t011	V
18CS002U	Primary screening	t011	V
18CS002U-F	Follow-up	t011	V
ATCC 43300			

Figure S2. Rep-MP3 PCR and RAPD fingerprints of MRSA isolates obtained from the follow-up investigation of farm workers. The dendrogram was generated with BioNumerics (Applied Maths) using the unweighted pair-group method with arithmetic averages (UPGMA) and the Dice coefficient.
